# Claudin-4 Undergoes Age-Dependent Change in Cellular Localization on Pig Jejunal Villous Epithelial Cells, Independent of Bacterial Colonization

**DOI:** 10.1155/2015/263629

**Published:** 2015-04-08

**Authors:** J. Alex Pasternak, Coral Kent-Dennis, Andrew G. Van Kessel, Heather L. Wilson

**Affiliations:** ^1^Vaccine and Infectious Disease Organization (VIDO), Home of the International Vaccine Centre (InterVac), 120 Veterinary Road, University of Saskatchewan, Saskatoon, SK, Canada S7N 5E3; ^2^Department of Animal and Poultry Science, University of Saskatchewan, 51 Campus Drive, Saskatoon, SK, Canada S7N 5A8

## Abstract

Newborn piglets are immunologically naïve and must receive passive immunity via colostrum within 24 hours to survive. Mechanisms by which the newborn piglet gut facilitates uptake of colostral cells, antibodies, and proteins may include FcRn and pIgR receptor-mediated endocytosis and paracellular transport between tight junctions (TJs). In the present study, FcRn gene (FCGRT) was minimally expressed in 6-week-old gut and newborn jejunum but it was expressed at significantly higher levels in the ileum of newborn piglets. pIgR was highly expressed in the jejunum and ileum of 6-week-old animals but only minimally in neonatal gut. Immunohistochemical analysis showed that Claudin-5 localized to blood vessel endothelial cells. Claudin-4 was strongly localized to the apical aspect of jejunal epithelial cells for the first 2 days of life after which it was redistributed to the lateral surface between adjacent enterocytes. Claudin-4 was localized to ileal lateral surfaces within 24 hours after birth indicating regional and temporal differences. Tissue from gnotobiotic piglets showed that commensal microbiota did not influence Claudin-4 surface localization on jejunal or ileal enterocytes. Regulation of TJs by Claudin-4 surface localization requires further investigation. Understanding the factors that regulate gut barrier maturation may yield protective strategies against infectious diseases.

## 1. Introduction

*In utero*, the pig fetus does not share circulation with the sow and therefore piglets are born immunologically naïve (i.e., without a complement of maternal antibodies). As a result, piglets must ingest colostrum within the first day of life or they will die from infectious diseases. In addition, piglets are born hypoproteinemic and require rapid maturation of the serum protein profile [1]. Colostrum-derived immunoglobulins (Igs) and other macromolecules (such as albumin, cytokines, and antimicrobial peptides, as well as many other bioactive products) traverse the gut wall then enter into the vasculature where they play a variety of roles including passive protection against disease [1–3]. There are several proposed mechanisms by which Igs and other macromolecules are absorbed by the gut wall but the primary mechanism in the newborn piglet is through nonselective pinocytosis by fetal-derived enterocytes [4–6] until cellular replacement occurs (approximately 19 days after birth [7]). “Gut closure” is defined as the “time after which intestinal epithelial cells no longer take up or internalize macromolecules via pinocytosis” [4] but although the upper half of the small intestine undergoes “gut closure” much earlier than the lower half of the small intestine, both the upper and lower regions of the small intestines lose their capacity to transport macromolecules to the blood at approximately two days of age [4, 5, 8]. Lecce (1973) further speculated that dietary-management regimen had a profound effect on the capacity of the neonatal gut epithelium to absorb and transport macromolecules as piglets starved for three days after birth continued to transport internalized macromolecules into the blood in a manner similar to the one-day-old piglet, but fed piglets did not [4]. However, this effect may have been mediated by the stress and inflammation induced by starvation rather than a direct impacted gut permeability [9]. Others showed that the capacity for the transmission of macromolecules was higher in low birth weight piglets (<1 kg) and that insulin may play a role in initiation of gut closure [10, 11].

Beyond pinocytosis, maternal IgG can be bound by the FcRn protein on the apical surface of the epithelial cells whereupon IgG-FcRn are endocytosed, trafficked to the basolateral surface, and released to the* lamina propria *in a pH-dependent manner [12]. Igs bound by FcRn are protected from proteolysis by being trafficked away from the lysosomal pathway [13] and back to the plasma membrane [14] where the elevated extracellular pH results in dissociation from FcRn. In pigs, humans, and nonhuman primates, FcRn is present on the intestine in adulthood and, therefore, may be a mechanism for antibody-mediated sampling of lumenal contents and uptake beyond the uptake of maternal antibodies in the neonate [15–17]. In contrast, rodent intestinal epithelial cells do not express FcRn after weaning [18]. Another mechanism of antigen uptake across the gut wall may occur via the pIgR receptor. Gut-derived plasma cells home to the mammary gland and secrete dimeric SIgA into the colostrum/milk, via pIgR transport [19, 20]. Upon ingestion of colostrum/milk by the neonate, SIgA may be absorbed by the gut through binding to pIgR possibly with an antigen in tow which can be transported to the circulation [21, 22]. Circulating or mucosal IgA may be subsequently transported from the basolateral to apical side of the gut mucosa via pIgR [21, 23]. Other mechanisms of macromolecular uptake across the gut wall include (1)* lamina propria* dendritic cell sampling of lumenal antigens by extension of their processes between epithelial cells whilst maintaining barrier integrity through the expression of tight junction (TJ) proteins [24] and (2) uptake across the characteristic follicle-associated epithelium (FAE) containing “microfold” (M) cells [25]. These specialized thin epithelial cells transfer effectively soluble, and especially particulate, antigens such as microorganisms from the lumen to dendritic cell [26]; (3) further, at least in mice, there is evidence that goblet cells deliver luminal antigen to dendritic cells in the small intestine [27]. Thus, there are multiple mechanisms by which antigen can traverse the gut wall.

A single layer of epithelial cells separates the apical and basolateral domains of the gut mucosa, and intercellular transport is regulated by complexes of TJ proteins, adherens, and desmosomes. Of these protein complexes, TJ proteins are located at the most apical side and play a central role in regulating permeability through the intercellular space within epithelial sheets [28–30]. TJs are composed of numerous structural and functional proteins including occludin and Claudin family members [31, 32] which together form a selectively permeable intercellular barrier [33]. Claudin family members have different expression pattern depending on cell type, location, and age, which may not be conserved across species [34–37]. Claudin-2, Claudin-3, and Claudin-4 have been detected in rat intestine [37, 38] and Claudin-1 to Claudin-4, Claudin-7 to Claudin-13, Claudin-15, and Claudin-18 have been detected in murine intestine [36]. Claudin-5 was initially attributed to be an endothelium-specific TJ protein [38, 39] but it has been specifically identified as an epithelial TJ protein as well [36, 37, 40]. Further, there are “tightening” Claudins (such as Claudin-1, Claudin-3, Claudin-4, and Claudin-5) [30, 40–42] as well as Claudins which meditate paracellular permeability for cations (such as Claudin-2 and Claudin-12) [37, 43]. Finally, mutations or changes in expression or surface localization of TJ proteins may lead to changes in intestinal permeability [30, 42, 44]. For instance, Bergmann et al. (2013) showed that mouse pups stressed for 12 hours showed increased intestinal permeability coincident with translocation of Claudin-4 from the region of the TJ on the surface of villous epithelial cells to the cytoplasm [42]. Age and environmental factors, at least in rodents, clearly impact epithelial cell surface localization of TJ proteins. Whether piglets, which are much more precocious at birth, also experience transitioning of TJ protein expression with age and region of the gut has not been elucidated.

We intend to establish whether there are regional and/or age-specific differences in the expression patterns of genes for FcRn, pIgR, Claudin-4, and Claudin-5. Florescent immunohistochemistry is used to establish patterns of Claudin-4 and Claudin-5 surface localization within distinct regions in the pig intestine over time to determine whether their surface localization changes are coincident with changes in intestinal permeability as the newborn gut matures. Claudin-4 and Claudin-5 were selected as a representative “tightening” TJ proteins found on intestinal epithelial cells and blood vessel endothelial cells, respectively. Because the gut of the newborn is “sterile” and microbiota contributes to maturation of the gut [45–48], we further investigated the role of commensal microbiota on tight junction protein surface localization.

## 2. Materials and Methods

### 2.1. Animal Use and Ethics and Description

This work was approved by the University of Saskatchewan's Animal Research Ethics Board and adhered to the Canadian Council on Animal Care Guidelines for humane animal use.

Conventionally raised Landrace cross piglets were obtained from the Prairie Swine Centre, Inc., Saskatoon, SK, Canada, and piglets within the 6 weeks of age group were weaned at 28 days of age.

Derivation of germ-free piglets, preparation of isolators, and experimental conditions for these piglets have been previously published [46]. Briefly, 16 piglets (>800 g of BW, Large White × White Duroc) were allocated to 4 treatment groups (*n* = 4/treatment) including piglets that remained germ-free (GF). Two groups were bottle-fed milk containing either 2 mL of 10^8^ colony-forming units (CFU)/mL nonpathogenic* Escherichia coli *(EC) or 2 mL 10^9^ CFU/mL with* Lactobacillus fermentum *(LF) (cultured from feces from a healthy sow as detailed in [46]) at 24 h and 30 h after birth. The bacterial inoculants were isolated from the cecum of a healthy adult sow, cultured for 18 hours at 37°C in a tryptic soy broth (BBL, Sparks, MD), and a subsample from each culture was taken for enumeration. Bacterial inoculants were typed to species level by sequencing of the chaperonin-60 universal target gene and query of cpnDB (http://www.cpndb.ca/cpnDB/home.php) [50]. Pigs in the conventional isolator were also fed at 24 hours and 30 hours after birth but their milk contained 2 mL of each of the monoassociated inoculants and 2 mL of fresh feces (obtained from a conventionally reared sow at Prairie Swine Centre Inc. mixed 1 : 1 with sterile phosphate-buffered saline (0.01 M phosphate, 0.15 M NaCl; pH 7.4)). This latter group is referred to as the sow feces (SF) group. Collectively, we will refer to the GF, EC, LF, and SF groups as the “gnotobiotic piglets.” As described in Shirkey et al. (2006), all piglets were fed to satiety at 3-hour intervals for the first 24 hours after birth with sterile-filtered porcine serum (Gibco, Burlington, Canada) mixed 1 : 1 with Similac (Abbott Laboratories, Abbott Park, IL) [46]. On Day 1, pigs were trough-fed a mixture of 2 : 1 Similac water (4.7 g/100 mL protein; 12.2 g/100 mL lipid; 24.3 g/100 mL carbohydrate)* ad libitum*. Pigs were fed milk in troughs at eight-hour intervals for the remainder of the trial.

### 2.2. Tissue Collection

From conventionally raised piglets, piglets were humanely killed by captive bolt and exsanguinated. We obtained tissues from 24-hour-old (*n* = 5) and 6-week-old (*n* = 5) piglets for gene expression analysis. A 10-cm small segment of Peyer's patch-free jejunum and ileum was excised, sliced into smaller fractions, immediately snap-frozen in liquid nitrogen, and then stored at −80°C until RNA extraction. Immunohistochemistry (IHC) was performed on tissues from piglets that are 24 hours old (*n* = 3), 48 hours old (*n* = 3), 3 days old (*n* = 3), 5 days old (*n* = 3), and 6 weeks old (*n* = 3). Tissues were fixed in 10% buffered formalin (Sigma-Aldrich, Oakville, ON, Canada) for 48 hours and then processed and embedded in paraffin by Prairie Diagnostic Services, University of Saskatchewan.

From gnotobiotic piglets, as published in [46], piglets (*n* = 4 per group) were removed from the isolators at 14 days of age, weighed, and killed by submersion in CO_2_ and exsanguinated. The small intestine was carefully dissected from the mesentery and its length was recorded. A 2 cm segment obtained at 50% (jejunum) and 95% (ileum) of the small intestinal length was placed in 10% buffered formalin for 24 hours before being transferred to 70% ethanol and embedded in paraffin.

### 2.3. Primer Design

Real-time primer sets for Claudin-4 gene (CLDN4), Claudin-5 gene (CLDN5), FcRn gene (FCGRT), pIgR gene (PIGR), and three stable reference genes (ACTB, HPRT, and RPL19) were designed using Primer3 software based on sequence data obtained from the National Center for Biotechnology Information (NCBI; http://www.ncbi.nlm.nih.gov/) (Table 1). Where possible, primers were designed to span exon-exon junctions as identified by BLAST like alignment tool (BLAT) comparison with SusScrofa10.2 genomic build. The primer sets were further verified for dimer and hairpin formation using OligoAnalyser v3.1 (Integrated DNA Technologies (IDT); http://www.idtdna.com/pages/scitools/) and target specificity was confirmed using the basic logical alignment search tool (BLAST) against the NCBI nucleotide database. The PCR efficiency for the primer probe set was evaluated against a serial dilution of pooled samples and found to be greater than 95% for all genes. Data was normalized to the geometric mean of the reference genes and statistical analysis was carried out on ΔCt values.

### 2.4. RNA Extraction and Gene Expression Data Analysis Using Real-Time Quantitative PCR

Gastrointestinal jejunum and ileum (*n* = 5 biological replicates per age group) were ground using a mortar and pestle and total RNA was isolated from using Trizol Reagent (Life Technologies, Carlsbad, CA, USA) as per the manufacturer's instructions with the addition of a second isopropanol (Commercial Alcohols, Inc., Brampton, ON, Canada) precipitation to completely remove phenol and other contaminants. DNA contamination was removed using the DNA-free kit (Life Technologies) before RNA quantity was determined on a NanoDrop spectrophotometer ND-1000 (NanoDrop, Wilmington, DE USA). RNA integrity was then evaluated on a 1.2% (w/v) denaturing agarose gel (Life Science Research Division, Bio-Rad Laboratories (Canada) Ltd., Mississauga, ON, Canada) to ensure that all samples had clear 28S and 18S ribosomal RNA banding patterns before they were carried forward. Reverse transcription (RT) was performed on 2 *μ*g of total RNA using the High Capacity cDNA Reverse Transcription Kit (Life Technologies) as per manufacturer's instructions. Sample cDNA was then diluted in nuclease-free water to 10 ng/*μ*L equivalent cDNA. Quantitative real-time polymerase chain reaction (qPCR) was performed in duplicate using 20 ng of equivalent cDNA, Kappa Fast Universal Mastermix (Kapa Biosystems, Wilmington, MA USA) with a primer concentration of 1 *μ*M using the IQ5 qPCR system (Bio-Rad, Hercules, CA, USA). Data is presented at the log normalized 2^−ΔΔCt^ form held relative to average expression for corresponding tissues from the 6-week-old animals.

### 2.5. Immunohistochemistry

Tissue sections were deparaffinised in xylene (Sigma-Aldrich) and rehydrated to distilled water in decreasing concentrations of ethanol (Commercial Alcohols, Inc). Heat-induced antigen-retrieval (HIAR) was carried out in Tris-EDTA buffer (10 mM Tris, 1 mM EDTA Solution, 0.05% Tween 20, pH 9.0; Sigma-Aldrich) for 30 min at 90°C. Slides were blocked for 3 hrs at room temperature in 5% (w/v) skim milk (Bio-Rad) in PBSA and then incubated overnight at 4°C with a 1 : 250 dilution of rabbit anti-CLDN4 (ab53156, Abcam, Cambridge, MA, USA) or 1 : 100 dilution of rabbit anti-CLDN5 (ab53765, Abcam) in incubation buffer (1% BSA, 1% Donkey Serum, and 0.5% Triton X-100 in PBS; Sigma-Aldrich). Slides were then washed three time in PBS and incubated in a 1 : 500 dilution of FITC-labeled goat anti-rabbit IgG (4030-02, Southern Biosystems, Birmingham, AL, USA) for anti-CLDN4 or PE-labeled goat anti-rabbit IgG (ab97070; Abcam) in incubation buffer at 4°C for 4 hours. Slides were again washed three times in PBS before the cover slip was added with Prolong Gold antifade with DAPI (Life Technologies). Intestinal villi were imaged using an Axiovert 200 M with a 63X neoFluor objective (Zeiss, Oberkochen, Germany) under oil immersion. Finally, DAPI and FITC/PE images were background subtracted and merged using ImageJ software [51].

### 2.6. Statistical Analysis

All statistical analyses and graphing were performed using GraphPad Prism 5 software (GraphPad Software, San Diego, CA, USA). As outcome variables were found to not be distributed normally, differences among all groups were examined by using a Wilcoxon matched-pairs signed rank test. Differences were considered significant if *P* < 0.05. (^∗^ *P* < 0.05, ^∗∗^ *P* < 0.01, and ^∗∗∗∗^ *P* < 0.0001).

## 3. Results and Discussion

### 3.1. Gene Expression Analysis on Jejunal and Ileal Tissues

**Figure 1:**
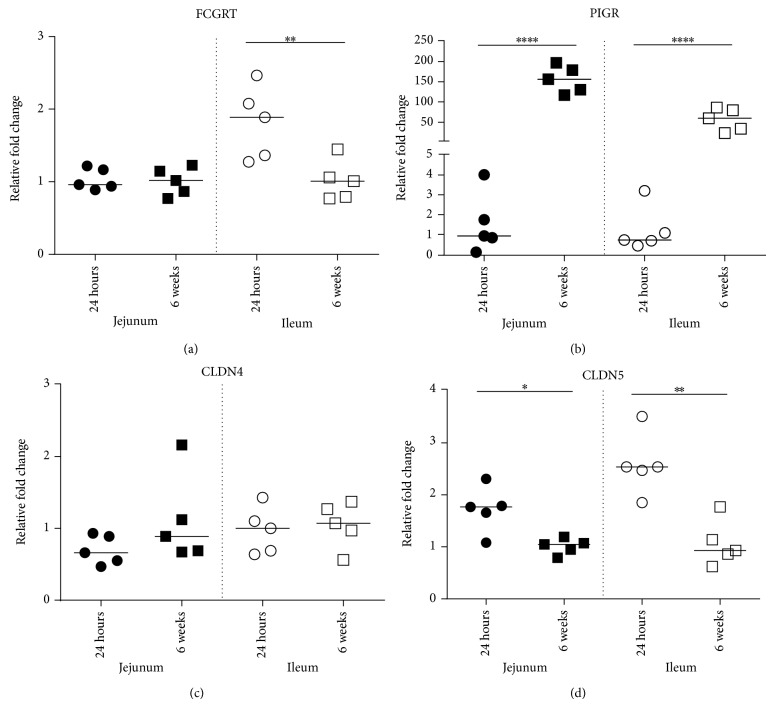
QPCR analysis of CLDN4 and CLDN5 expression in jejunal and ileal gut tissue. The mRNA expression levels of FCGRT, PIGR, CLDN4, and CLDN5 genes were normalized with the reference genes and were calculated with 2^−ΔΔCt^ relative quantification. Dots show the data for each biological replicate (*n* = 5 per group). Horizontal bars represent the median values (^∗^ *P* < 0.05; ^∗∗^ *P* < 0.01; ^∗∗∗∗^ *P* < 0.0001).

To determine whether FCGRT, PIGR, CLDN4, and CLDN5 gene expression changed with age and/or showed differential expression depending on the region of the gut under investigation, qRT-PCR analysis was performed on segments of jejunum and ileal gut tissue from piglets that were 24 hours old (*n* = 5) or 6 weeks of age (*n* = 5). As expected, the gene expression patterns for the antibody binding receptors FcRn and pIgR were strikingly different. Tissues from 24-hour-old piglets and 6-week-old pigs showed equivalent expression levels of FCGRT in the jejunum while, in the ileum, 24-hour-old piglets showed a statistically significant induction of FCGRT over that of 6-week-old animals (Figure 1(a); *P* < 0.01). pIgR, on the other hand, was expressed at minimal levels in both the neonatal jejunum and ileum relative to that of jejunal and ileal-derived tissues from the 6-week-old pigs (Figure 1(b); *P* < 0.0001). Of course, it is not surprising that pIgR levels are high in 6-week-old piglets as this receptor would be required to translocate piglet-derived SIgA to the lumen. We simply point out that, because the PIGR is minimally expressed on newborn piglets' jejunal or ileal enterocytes, colostrum-derived SIgA would not be taken across the gut wall through binding and receptor-mediated endocytosis of the pIgR.

With regard to the expression transcripts for TJ proteins, CLDN4 was expressed at equivalent levels in the jejunum and ileum from 24-hour-old piglets relative to corresponding tissues in the 6-week-old animals with no statistically significant difference (Figure 1(c)). Statistically, less CLDN5 mRNA was expressed in the jejunum (Figure 1(d); *P* < 0.05) and ileum (*P* < 0.01) in the 6-week-old animals compared to the region-specific tissues obtained from animals that were 24 hours old (Figure 1(d)). As with CLDN4, the median values for CLDN5 expression from each region of the gut were highly conserved across both tissues for each age group.

### 3.2. Evaluation of Claudin-4 Surface Localization on Jejunal and Ileal Enterocytes

**Figure 2:**
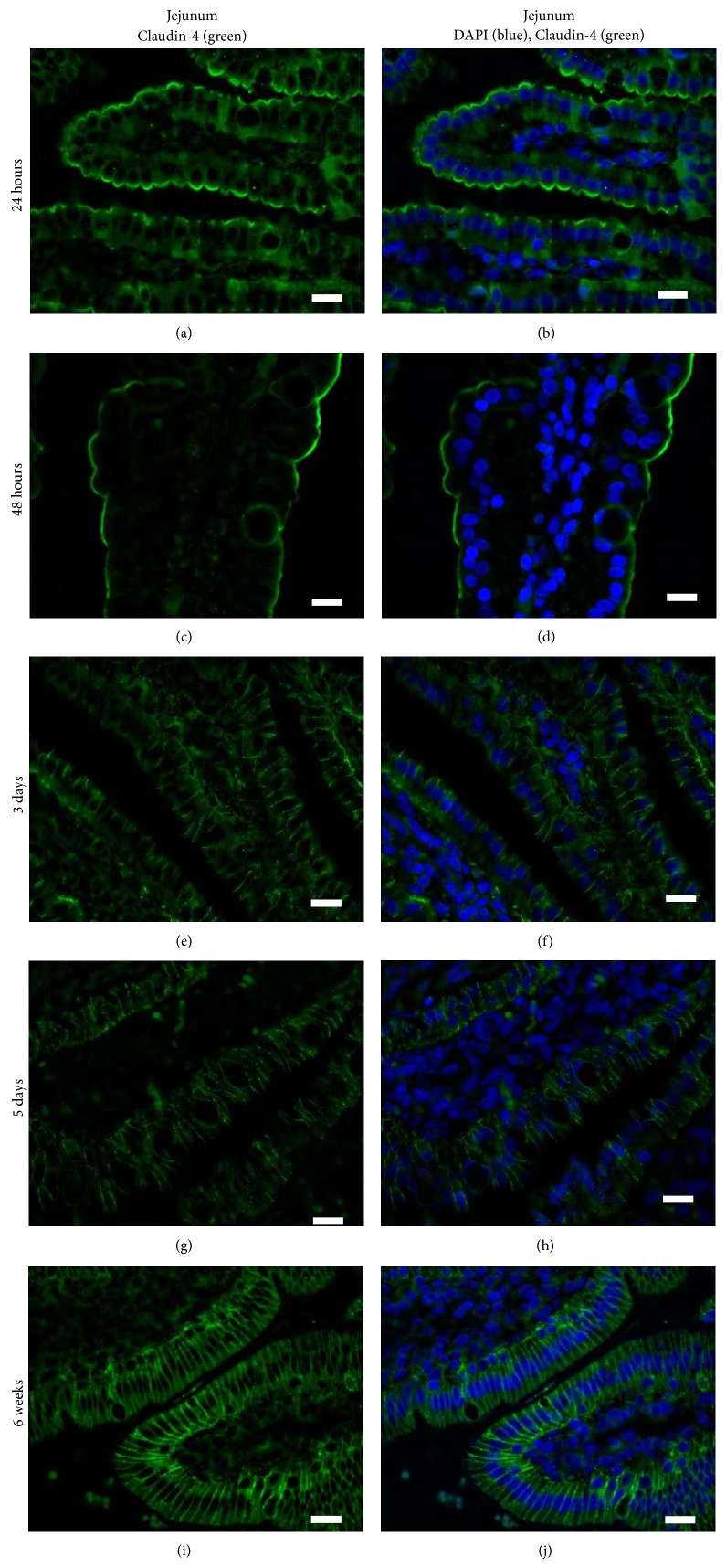
Claudin-4 surface localization changes over time in piglet jejunum. Claudin-4 is localized to the apical aspect of the surface of jejunal intestinal enterocytes at 24 hours of age ((a), (b)) and at 48 hours of age ((c), (d)). At 3 ((e), (f)) and 5 ((g), (h)) days of age, Claudin-4 is localized along the cellular surface between the adjacent cells and no longer at the apical aspect of the enterocytes. At 6 weeks of age, there is very strong expression at the lateral membranes between adjacent enterocytes ((i), (j)). These images are representatives of IHC performed on tissue from 3 animals per time point. Primary antibody: rabbit anti-Claudin-4. Secondary antibody: FITC-labeled goat anti-rabbit (green). Nuclear stain: DAPI (blue).

**Figure 3:**
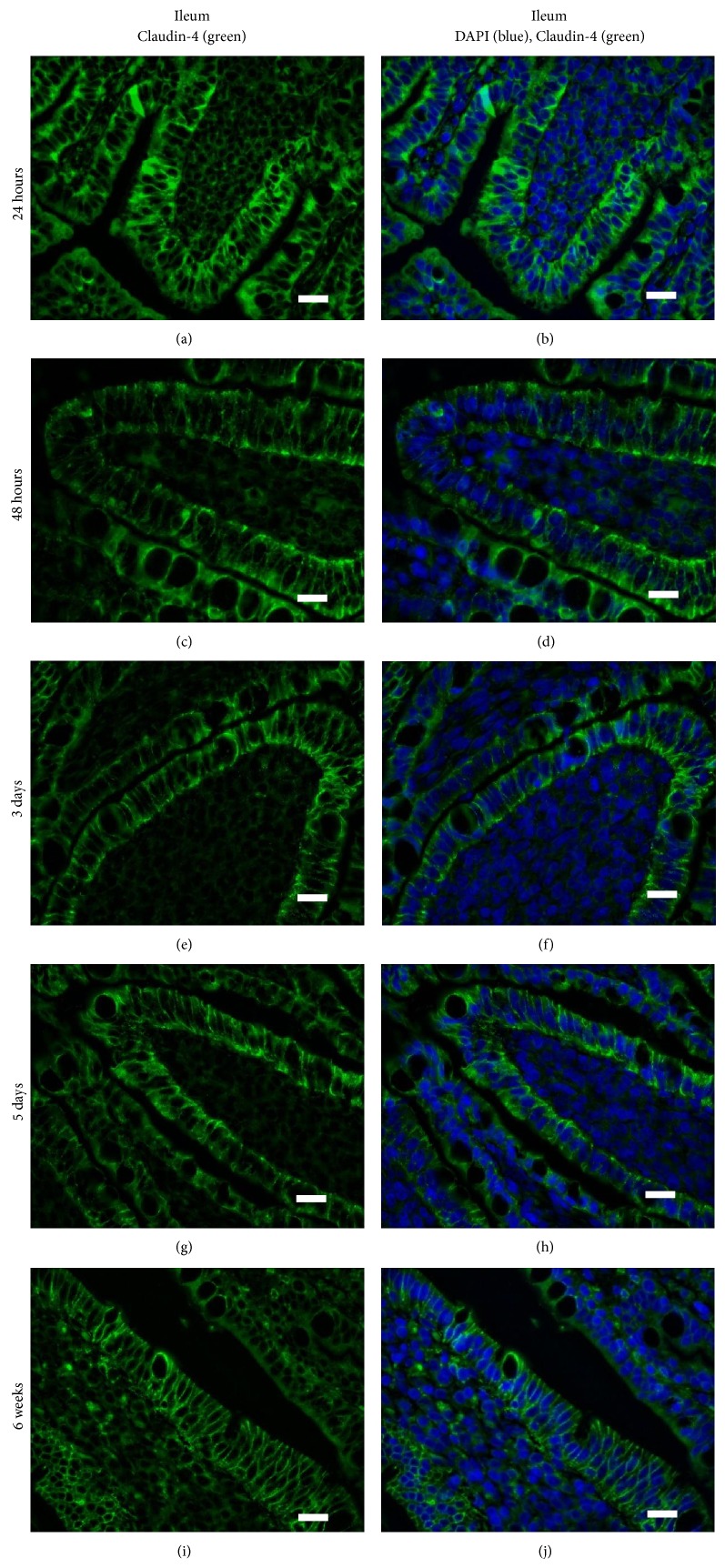
Claudin-4 surface localization does not change over time in piglet ileum. Claudin-4 is highly expressed on the surface of ileal intestinal enterocytes with the highest expression on the surfaces between adjacent cells at 24 hours of age ((a), (b)), 48 hours of age ((c), (d)), 3 days of age ((e), (f)), 5 days of age ((g), (h)), and 6 weeks of age ((i), (j)). These images are representatives of IHC performed on tissue from 3 animals per time point. Primary antibody: rabbit anti-Claudin-4. Secondary antibody: FITC-labeled goat anti-rabbit (green). Nuclear stain: DAPI (blue). Scale bar represents 40 *μ*m.

Different TJ family members localize to distinct regions (i.e., on the crypts or the villi) and, at the cellular level, they can be expressed along the lateral surface between adjacent cells or preferentially on the apical or basolateral surfaces. Tamagawa et al. (2003) showed that, in the mouse (age not specified), Claudin-2 was present within the crypts of the small and large intestine, Claudin-3 was present in both the villi and crypts, and Claudin-4 was only modestly associated with the villous tips of the small and large intestine [52]. Within these regions, Claudin-2 and Claudin-3 were localized to the apical surfaces of the intestinal epithelial cells but Claudin-4 appeared to be highly localized to FAE dome of the mouse intestine, which the authors speculate may regulate intercellular junctions to allow antigen sampling by dendritic cells [52]. Patel et al. (2012) showed that Claudin-3 localized to the TJs of crypt epithelium in 2- day-old and 2-week-old mice and transitioned to being also localized to the TJs in villous epithelium at three weeks of age when the murine gut is mature [53]. The kinetics of Claudin protein expression and surface localization may correlate with changes in gut permeability. Corroborating this hypothesis, Bergmann et al. (2013) showed that mouse pups stressed for 12 hours showed increased intestinal permeability coincident with translocation of Occludin and Claudin-4 from the region of the TJ on the surface of villous epithelial cells to the cytoplasm [42]. We used IHC to investigate whether there were age-specific or region-specific changes in Claudin-4 surface localization in pig intestinal epithelial cells. We observed that at 24–48 hours of age, Claudin-4 (green) was localized to the extreme apical aspect of the jejunal enterocytes along the villi and the crypts (only villi shown; Figures 2(a)–2(d)). During these times, very little if any Claudin-4 protein was present at the region of the cell where the TJs are formed. From tissues obtained from piglets that were 3 and 5 days of age, however, we clearly see that Claudin-4 was highly localized on the lateral surface of the cells in contact with adjacent enterocytes (Figures 2(e), 2(f), 2(g), and 2(h)). At 6 weeks of age, Claudin-4 level of expression appears even stronger than at 3 and 5 days of age and it is still localized along the cell surface adjacent to other enterocytes (Figures 2(i) and 2(j)). In stark contrast, when we investigated the surface localization pattern of Claudin-4 on ileal intestinal epithelial cells, we observed that, even at 24 hours of age (Figures 3(a) and 3(b)), Claudin-4 was localized along surface of the cells adjacent to other enterocytes (including the region of the TJs) and this pattern remained unchanged with age (Figures 3(c)–3(j)).

Thus, our data shows that, at 24 and 48 hours of age (Figures 2(a)–2(d)), Claudin-4 was localized to the apical surface of the jejunal enterocytes which coincides with the period of time in which the piglet jejunum is permeable to both maternal Ig and other macromolecules as well as maternal cells which, after ingestion, are reported to be quickly found in the circulation [4, 5]. Between 48 hours of age and 3 days of age, Claudin-4 relocated along the surface of the cells that are immediately adjacent to neighbouring epithelial cells which coincides with the time that jejunal enterocytes cease to absorb macromolecules. We believe it is unlikely that jejunal enterocytes at 3 days of age represent “nonfetal” derived cells as the fetal enterocytes are reported to be replaced after 2-3 weeks [7]. However, more studies will need to be performed to verify that it is indeed fetal-derived enterocytes, which show a change in Claudin-4 surface localization. Claudin-4 is known to be a “tightening” Claudin [42] and the fact that it localizes to the tight junction region in the jejunum at the same time these cell lose their capacity to absorb macromolecules may reflect a mechanism of decreasing gut permeability [4]. Williams (1993) determined that, in colostrum-deprived piglets that were less than 4 hours old, ingested FITC-labeled colostral leucocytes penetrated the jejunal epithelium (in which our data indicates that Claudin-4 is not yet at the site of TJs at this time) but not the ileum (in which our data indicates that Claudin-4 is at the site of TJs within 24 hours after birth) [54]. However, at this time, we cannot say whether events are related or simply coincidental. In future studies, confocal microscopy will be used to establish whether jejunal or ileal enterocytes show a change in localization of Claudin-4 from the cytoplasm to the surface, which may contribute to changes in gut permeability. Further, we cannot find any studies which determine how long after birth maternal colostrum-derived cells can traverse the gut wall. If they can cross the gut wall after one week of age, for example, then it is unlikely that Claudin-4 surface localization to the region of tight junctions regulates maternal cell uptake. However, if maternal cells cannot traverse the gut wall after 2 days of age, then perhaps regulation of Claudin-4 surface localization may mediate cellular uptake. These studies may have important implications for pig husbandry as piglets are routinely cross-fostered and therefore do not have continuous access to colostrum from their dams.

### 3.3. Evaluation of Claudin-5 Surface Localization on Jejunal and Ileal Blood Vessel Endothelial Cells

**Figure 4:**
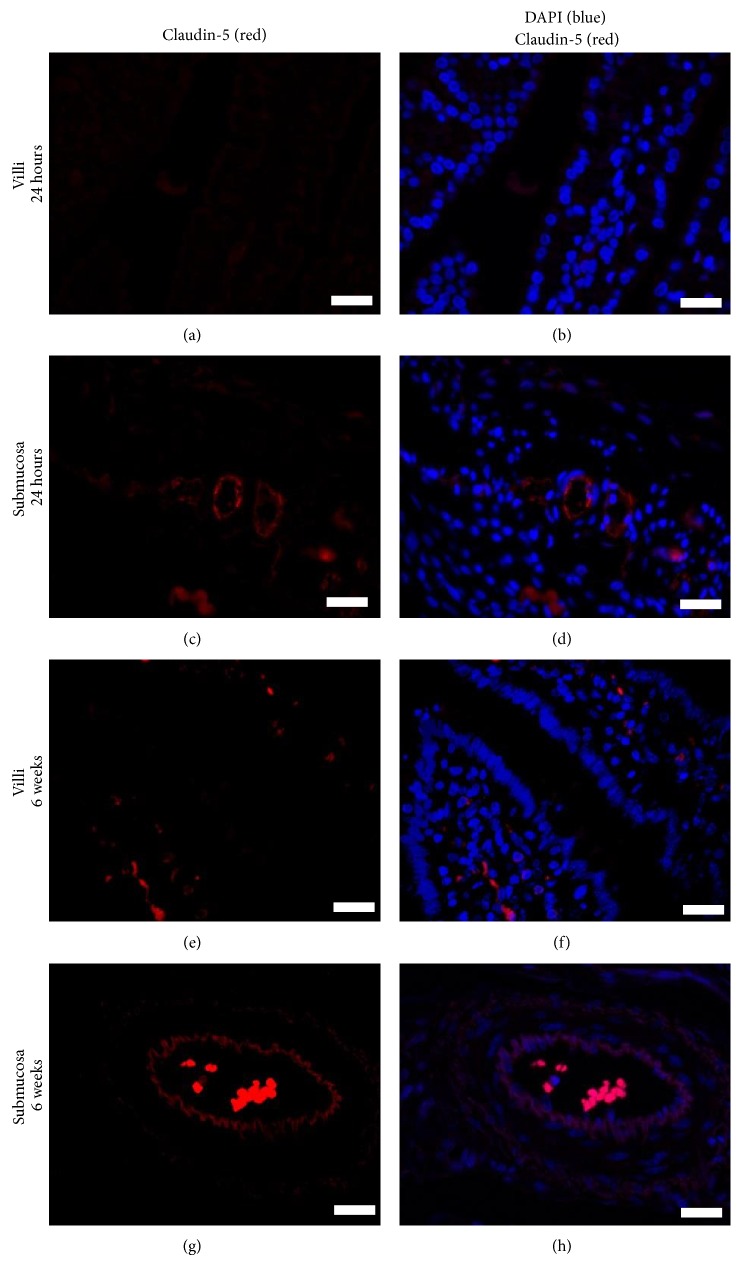
Claudin-5 surface localization on blood vessel endothelial cells does not change over time. Claudin-5 is absent from the surface of jejunal enterocytes in 24-hour-old ((a), (b)) and 6-week-old piglets ((e), (f)), respectively but it is present on the blood vessel walls in the submucosa for both age groups ((c), (d), (g), and (h)). Red cells within villi are from autofluorescent cells and red cells within the blood vessels are red blood cells. These images are representatives of IHC performed on tissue from 3 animals per time point. Primary antibody: rabbit anti-Claudin-5. Secondary antibody: PE-labeled goat anti-rabbit (red). Nuclear stain: DAPI (blue). Scale bar represents 40 *μ*m.

CLDN5 is a TJ-regulating gene on epithelial cells and endothelial cells which plays a role in regulating intestinal and vascular permeability [36, 37, 40, 55, 56]. We next investigated whether Claudin-5 was expressed on piglet intestinal epithelial cells and the endothelial cells that line the blood vessel walls. IHC was performed on jejunal villi and blood vessels in the submucosa. In contrast to studies in rats and mice where Claudin-5 was shown to be expressed on intestinal epithelial cells, Claudin-5 was not expressed on the surface of jejunal or ileal villous epithelial cells (Figures 4(a) and 4(b)) in the piglet, but it was expressed on the blood vessel endothelial cells from this age group (Figures 4(c) and 4(d)). In a similar fashion, Claudin-5 was absent from the villi on the 6-week-old animals (Figures 4(e) and 4(f)) but it was present on their blood vessel endothelial cells (Figures 4(g) and 4(h)). (The red fluorescent cells in the lamina propria (Figures 4(c) and 4(d)) are autofluorescent cells and the red fluorescent cells within the blood vessel (Figures 4(g) and 4(h)) are red blood cells.) Our data shows that Claudin-5 surface localization on endothelial cells does not change with age and likely is not critical for regulating macromolecule transport into blood vessels as the piglet gut matures. It may be that another TJ protein besides Claudin-5 fulfills this role or it may be that paracellular leakage is not the method by which cells or macromolecules absorbed by jejunal or ileal epithelial cells enter into the neonatal blood stream. Vascular permeability assays or transendothelial leukocyte migration assays may provide insight into whether the blood vessels are transiently permeable in the neonatal piglet gut prior to 2 days of age.

We recognize that it would be of significant interest to characterize the surface expression of many other claudin family members as well as members of the zonodulin, occludin, and junction adhesion molecule families. We evaluated several commercial antibodies, which were indicated to be cross-reactive with pig but, despite using several antigen-retrieval methods, only Claudin-4 and Claudin-5 were successful for use in IHC in our hands (data not shown).

### 3.4. Commensal Microbiota Does Not Influence Claudin-4 Surface Localization

**Figure 5:**
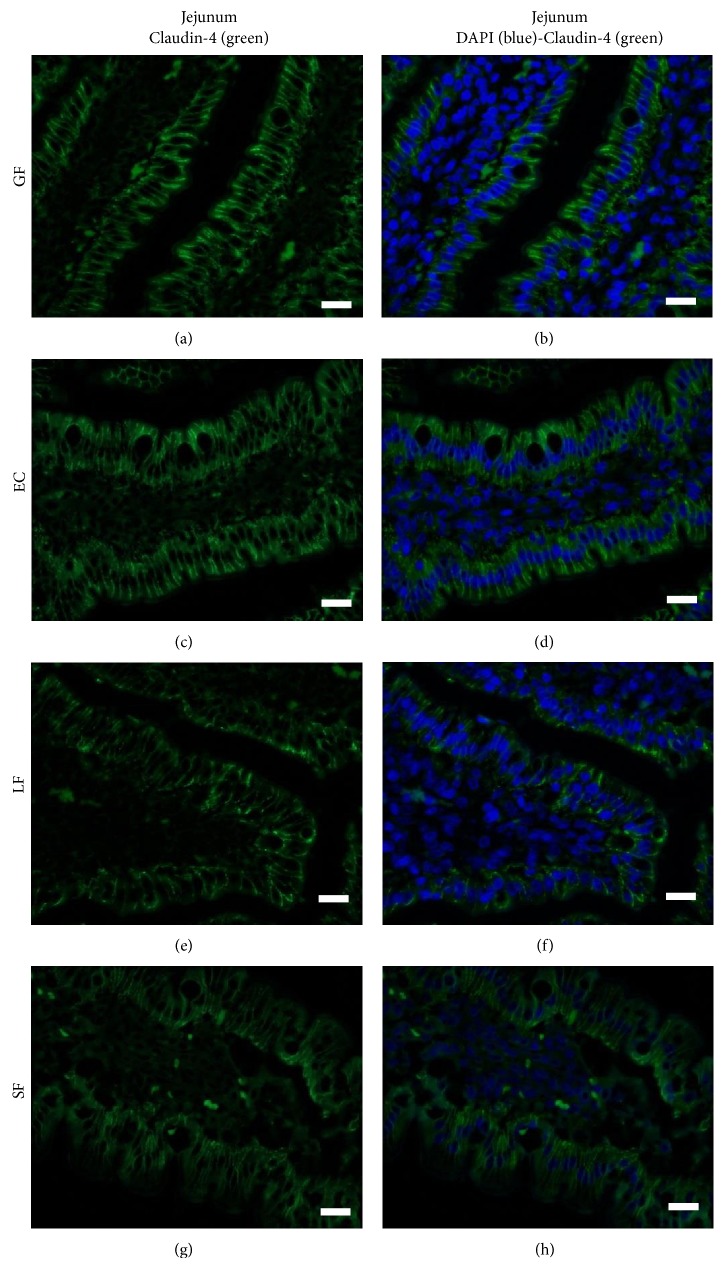
Claudin-4 surface localization in the piglet jejunum is not influenced by colonization by commensal microbiota.In the jejunum of 14-day-old germ-free piglets ((a), (b)), piglets colonized with EC ((c), (d)), LF ((e), (f)), or conventional microbiota (SF; (g), (h)), Claudin-4 is localized to the surface between adjacent enterocytes. These images are representatives of IHC performed on tissue from 4 animals per time point. Primary antibody: rabbit anti-Claudin-4. Secondary antibody: FITC-labeled goat anti-rabbit (green). Nuclear stain: DAPI (blue). Scale bar represents 40 *μ*m.

The newborn intestine is sterile at birth, but it quickly becomes colonized with microbes derived from the maternal birth canal and the external environment.* E. coli* and other coliforms are the earliest colonizers of the pig digestive tract followed by* Clostridium* and* Lactobacillus* species which supplant* E. coli* as the dominant isoform within 48 hours [57, 58]. We previously reported that early colonizing nonpathogenic* E. coli* and* Lactobacillus fermentum* differentially affect villous structure [46], enterocyte turnover [59], and intestinal maturation specifically pertaining to digestive function [45]. Here, we wanted to determine whether Claudin-4 surface localization on gut epithelial cells was influenced by colonization of the piglet gut with commensal flora. We compared intestinal Claudin-4 surface expression on the villi from piglets raised for 14 days as GF animals, animals monocolonized by* EC* or* LF* or colonized with microbiota from sow feces (SF) spiked with EC and LF. The gut segment at 50% of the length of the small intestine (previously shown to be devoid of ileal Peyer's patches (IPP) and therefore likely represent the jejunum) was chosen for examination [46]. Because Claudin-4 was already expressed in the regions of the TJs in ileal villous epithelium at 24 hours of age (Figures 3(a) and 3(b)), this region was not investigated in the gnotobiotic piglets. Regardless of whether piglets were raised GF (Figures 5(a) and 5(b)) or colonized with EC (Figures 5(c) and 5(d)), LF (Figures 5(e), and 5(f)), or SF (Figures 5(g) and 5(h)), the jejunal villi and crypts (crypt data not shown) showed comparable Claudin-4 surface localization on the region of the cells in contact with adjacent enterocytes. These data suggest that either Claudin-4 localization was not influenced by microbiota or, if it did have an impact, it was rectified by 14 days of age.

We find it intriguing that commensal microbiota did not appear to impact Claudin-4 surface localization in jejunum of 14-day-old gnotobiotic piglets. Studies in mice showed that although the gut is functionally mature at 3 weeks of age, mice treated for the first weeks of life with antibiotics exhibited decreased and immature expression of Claudin-3 and immature barrier function compared with control mice [53]. These authors go on to show that mice enterally administered live or heat-killed probiotic bacteria* Lactobacillus rhamnosus GG* showed significantly improved barrier function with decreased intestinal permeability [53]. These results indicate that, at least in mice, intestinal barrier function, as regulated by TJ protein expression, can be influenced by gut microbiota. Others showed that* B. infantis* positively impacted TJ formation and barrier-preserving properties [42]. Probiotic bacteria may act by normalizing microbial populations or by directly improving host defense mechanisms, specifically by strengthening intestinal barrier function, which, in turn, may reduce systemic entry of gut luminal microbes or toxins. These data suggest that, in mice, Claudin-3 and Claudin-4 may play a pivotal role in probiotic-induced barrier maturation [42]. Likewise, a study in seven-week-old dairy calves showed that calves fed milk replacer plus calf starter had different expression patterns for the genes coding for Occludin and Claudin-4 (but not Claudin-1) in the jejunum and the ileum when compared to calves fed milk replacer alone [60]. The authors speculate that these differences may be due to diet-specific bacterial diversity [60]. Using IHC, our data indicates that, in 14-day-old piglets, Claudin-4 localization to TJ of jejunal epithelial cells was not impacted by floral colonization.

Colostrum is rich in maternal leucocytes and it is estimated that piglets absorb several hundred million maternal cells daily [61, 62]. The mechanism of maternal cell uptake is not yet clear. Fluorescently labeled colostral leucocytes ingested by 2–4-hour-old, colostrum-deprived piglets penetrated the duodenal and jejunal epithelium, whereupon they migrated through the lymphatics and the peripheral blood and seeded nonlymphatic tissues, including mesenteric lymph nodes, spleen, liver, and lungs [54]. These cells were not absorbed across the ileum suggesting that the process is regionally selective [54]. Further, electron microscopy showed that radiolabeled, colostrum-derived lymphoid cells administered to piglets between 7 and 10 hours after birth were absorbed but if the cells were heat-treated, derived from the sow's blood, or obtained from another sow's colostrum, the cells did not traverse the gut wall, suggesting that the cells themselves may facilitate uptake [61]. Piglets born to vaccinated sows received sufficient* M. hyopneumoniae*-specific cell-mediated immunity to elicit significant delayed-type hypersensitivity responses three days after* M. hyopneumoniae* exposure, indicating that maternal cells taken up by the piglet are functional [63]. A study in calves showed that blood-derived PBMCs traversed the gut wall of 6-hour-old calves but only if the cells were incubated with acellular colostrum suggesting that factor(s) in colostrum promotes changes in the leucocytes which is necessary for uptake [64]. It is not yet clear how maternal cells traverse the gut wall but the above experiments suggest that colostrum-derived lymphoid cells play an active role in uptake and that the route may be through the paracellular route [61]. Future experiments will be performed to elucidate whether maternal cells and macromolecules indeed traverse the neonatal gut wall between adjacent enterocytes until Claudin-4 has relocated to the lateral surface of the cell. We will also use Ussing chambers to show that, upon Claudin-4 translocation to the lateral membrane, paracellular transport of labeled cells or macromolecules ceases. These experiments will provide indirect evidence that Claudin-4 is critically required for functional TJs.

## 4. Conclusions

Thus, our data indicates that, in the mixed cell populations that comprise jejunal and ileal tissue, FCGRT was minimally expressed in the ileum but showed higher expression in the jejunum from piglets less than 24 hours old. In contrast, PIGR gene expression showed a striking increase in both regions in the 6-week-old piglet gut relative to the tissues from the 24-hour-old piglets. CLDN4 and CLDN5 transcript abundance was conserved in jejunum and ileum in age-matched animals and striking differences in CLDN4 expression did not occur in either region of the gut with age. CLDN5 showed significantly higher expression in the jejunum and ileum from the 24-hour-old animals relative to the older animals. At the time period when the piglet gut is considered “leaky” (i.e., within the first two days of life) [4], jejunal enterocytes showed Claudin-4 protein localization at the apical aspect of jejunal enterocytes whereas ileal intestinal epithelial cells showed Claudin-4 localization at the surface associated with TJ formation. At 6 weeks of age, Claudin-4 was localized to lateral membranes in both the jejunum and ileum enterocytes. These data are intriguing as they provide evidence that Claudin-4 is not localized to the region of the jejunal TJs at the time when the jejunum is permeable. Microbiota did not impact Claudin-4 localization in 14-day-old piglets. More experiments are needed to directly establish whether Claudin-4 localization on jejunal villous epithelial cells directly impacts paracellular permeability in the neonatal piglet gut and thus impacts uptake and subsequent transport of maternal cells, antibodies, and other macromolecules into the blood.

## Figures and Tables

**Table 1:** Primer information.

Gene	Source	Sequence (5′-3′)	Amplicon size	Annealing temperature
ACTB	Nygard et al., 2007 [49]	Forward: CACGCCATCCTGCGTCTGGA Reverse: AGCACCGTGTTGGCGTAGAG	108	60

CLDN4	NM_001161637.1 (NCBI)	Forward: CAACTGCGTGGATGATGAGA Reverse: CCAGGGGATTGTAGAAGTCG	140	60

CLDN5	NM_001161636.1 (NCBI)	Forward: CCTTCCTGGACCACAACATC Reverse: CACCGAGTCGTACACCTTGC	110	60

FCGRT	NM_214197.2 (NCBI)	Forward: GTCTGGGAAAGCCAGGTGT Reverse: CCTCCTTCCTCCAAGGTTTT	104	60

HPRT	Nygard et al., 2007 [49]	Forward: GGACTTGAATCATGTTTGTG Reverse: CAGATGTTTCCAAACTCAAC	91	60

OCLN	NM_001163647.2 (NCBI)	Forward: GAGTACATGGCTGCTGCTGA Reverse: TTTGCTCTTCAACTGCTTGC	102	60

PIGR	NM_214159.1 (NCBI)	Forward: GCCAAGGTCCTGGACAGATA Reverse: GTACACGGATTTCGGCTTCT	116	60

RPL19	AF_435591 (NCBI)	Forward: AACTCCCGTCAGCAGATCC Reverse: AGTACCCTTCCGCTTACCG	147	60
